# Evaluation of *NTRK* Fusions Detection Method in Esophageal Squamous Cell Carcinoma and Gastric Adenocarcinoma

**DOI:** 10.3390/ijms27010336

**Published:** 2025-12-28

**Authors:** Tomoyuki Momma, Motonobu Saito, Shotaro Nakajima, Katsuharu Saito, Erika Machida, Ken Miyabe, Yusuke Sato, Hiroyuki Hanayama, Hirokazu Okayama, Zenichiro Saze, Kosaku Mimura, Naoto Tsuchiya, Akiteru Goto, Kouya Shiraishi, Koji Kono

**Affiliations:** 1 Department of Gastrointestinal Tract Surgery, Fukushima Medical University School of Medicine, Fukushima 960-1295, Japan; 2 Department of Cancer Genome Medicine, Fukushima Medical University Hospital, Fukushima 960-1295, Japan; 3 Department of Multidisciplinary Treatment of Cancer and Regional Medical Support, Fukushima Medical University School of Medicine, Fukushima 960-1295, Japan; 4 Division of Genome Biology, National Cancer Center Research Institute, Tokyo 104-0045, Japan; 5 Department of Surgery, Saitama Medical Center, Jichi Medical University, Saitama 330-8503, Japan; 6 Department of Cellular and Organ Pathology, Graduate School of Medicine, Akita University, Akita 010-8543, Japanakigoto@med.akita-u.ac.jp (A.G.); 7 Department of Esophageal Surgery, Akita University Hospital, Akita 010-8543, Japan; 8 Department of Blood Transfusion and Transplantation Immunology, Fukushima Medical University School of Medicine, Fukushima 960-1295, Japan; 9 Laboratory of Molecular Carcinogenesis, National Cancer Center Research Institute, Tokyo 104-0045, Japan; 10 Clinical Cancer Research Division, Shonan Research Institute of Innovative Medicine, Shonan Kamakura General Hospital, Kamakura 247-8533, Japan; 11 Department of Clinical Genomics, National Cancer Center Research Institute, Tokyo 104-0045, Japan

**Keywords:** *NTRK* fusions, esophageal squamous cell carcinoma, gastric adenocarcinoma

## Abstract

Neurotrophic tyrosine receptor kinase (*NTRK*) fusions function as oncogenes and have been targeted by TRK inhibitors with excellent clinical outcomes. The international expert consensus recommends immunohistochemical (IHC) screening for TRK protein followed by next generation sequencing (NGS) to measure expression of *NTRK* fusions for tumors with low *NTRK* fusion expression. To confirm the clinical utility of this recommendation in esophageal and gastric cancers, total TRK protein expression was measured by IHC using anti-pan-TRK antibody in 254 esophageal squamous cell carcinoma (ESCC) and 401 gastric adenocarcinoma (GA) samples. Subsequently, DNA-based NGS and fluorescence in situ hybridization (FISH) were performed for tumors expressing TRK to measure *NTRK* fusion expression. Further, expression of *NTRK* fusions was evaluated in esophageal and gastric cancers using public databases. IHC staining revealed TRK was expressed in 10 out of 254 ESCC and 0 out of 401 GC cases. NGS and FISH analyses were performed for 10 TRK positive ESCC cases, identifying that none of these cases harbored *NTRK* fusions. In silico analyses further confirmed that *NTRK* fusions are rarely present in esophageal and gastric cancers. IHC screening for TRK protein is recommended to detect *NTRK* fusions, but this method may include many false-positives cases based on the sequencing analysis.

## 1. Introduction

Neurotrophic tyrosine receptor kinase (*NTRK*) genes consist of three isoforms, *NTRK1*, *NTRK2*, and *NTRK3*, and encode a family of tropomyosin receptor kinases (TRK), including TRKA, TRKB, and TRKC, respectively [1]. TRK proteins are physiologically expressed in the central and peripheral nervous system, and comprise the receptor tyrosine kinase family of neurotrophin receptors [1]. Among gene aberrations occurring in *NTRK*, the most significant oncogenic aberration is gene fusion that continually activates TRK receptors, causing constitutive activation of downstream oncogenic signaling [1,2]. This oncogenic activation leads to phosphorylation of the TRK protein, and the efficacy of tyrosine kinase inhibiters (TKI) is widely recognized, along with *ALK*, *RET*, and *ROS1* fusions [3]. In *NTRK* fusion-positive cancers, larotrectinib and entrectinib have demonstrated excellent clinical outcomes [4,5].

While *NTRK* fusions are rarely detected in common cancers, they are highly prevalent in rare cancers, such as secretory breast carcinoma, mammary analog secretory carcinoma, adult and infantile congenital fibrosarcoma, and pediatric cellular and mixed congenital mesoblastic nephroma [1]. In gastrointestinal tract cancers, *NTRK* fusions are relatively highly detected in gastrointestinal stromal tumor (GIST), primarily in cases without *KIT*, *PDGFRA*, or *RAS* mutations, and in colorectal cancer, most frequently in Microsatellite Instability (MSI)-high tumors [1,6,7]. On the other hand, The Cancer Genome Atlas (TCGA) and FoundationCORE databases suggest *NTRK* fusions are rarely present in esophageal and gastric cancers [8,9].

To detect *NTRK* fusions, immunohistochemical (IHC) staining, fluorescence in situ hybridization (FISH), RT-qPCR, and both RNA- and DNA-based next generation sequencing (NGS) are performed [10,11]. Although NGS is the definitive means for detection of *NTRK* fusions, it is not feasible to routinely perform this advanced analysis in all patients. Therefore, the international expert consensus recommends IHC screening for TRK expression followed by NGS analysis to measure expression of *NTRK* fusions in TRK^+^ (positive) cases [10,12]. Importantly, the positivity rate of TRK staining is different between studies, possibly due to differences in patient demographics and antibodies for used IHC staining. For example, a prior report used TCGA data from Caucasian populations to identify that *NTRK* fusions were not detected in any of the analyzed esophageal squamous cell carcinoma (ESCC) or gastric adenocarcinoma (GA) cases [9]. In addition, very few ESCC or GA cases from Caucasian populations were identified as TRK^+^ by IHC staining with the anti-pan-TRK antibody [13,14]. Seemingly in contrast, other studies reported that 20–65% of GA cases from Asian populations expressed TRKs using IHC staining with anti-TRKA, TRKB, or TRKC antibodies [15,16]. Therefore, the practical utility of the consensus-recommended screening method must be further evaluated in esophageal and gastric cancers.

In the present study, we measured TRK positivity by IHC staining using a pan-TRK antibody in ESCC and GA samples from Asian populations (Japanese). Subsequently, NGS or FISH was performed on TRK^+^ cases to measure expression of *NTRK* fusions. We further discuss the utility of *NTRK* fusion screening methods for esophageal and gastric cancer in the context of present and prior findings.

## 2. Results

### 2.1. TRK Expression in ESCC and GA

**Figure 1:**
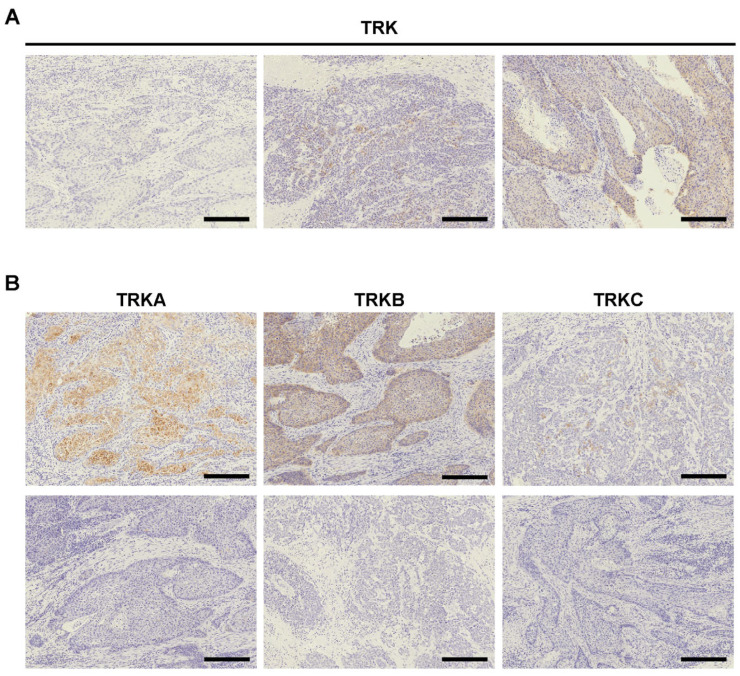
IHC for TRK, TRKA, TRKB, and TRKC in esophageal squamous cell carcinoma (ESCC). (**A**) Representative images showing immunohistochemical (IHC) staining with anti-pan-TRK antibody in ESCC. Negative TRK staining (left panel), weak positive (middle panel), and moderate positive (right panel) are shown. Scale bars = 250 μm. (**B**) Representative images showing IHC staining using anti-TRKA, anti-TRKB, and anti-TRKC antibodies in ESCC. Positive TRKA, TRKB, and TRKC staining (upper panels) and negative staining (lower panels) are shown. Scale bars = 250 μm.

We first performed IHC staining for TRK (pan-TRK), TRKA, TRKB, and TRKC in our cohort, which included 254 ESCC and 401 GA tumors (Figure 1, Table 1 and Table 2, and Supplemental Figure S1). The GA cohort included 27 (6.7%) EBV (+) cases and 33 (8.2%) dMMR GA cases. IHC staining for pan-TRK revealed that among ESCC cases, ten cases were weakly or moderately positive for TRK expression (Table 3). When comparing clinicopathological factors between TRK^+^ and TRK^−^ ESCC cases, no significant findings were observed. Contrastingly, no TRK^+^ cases were detected in the GA study cohort. IHC staining for TRKA, TRKB, and TRKC for the ten TRK^+^ ESCC cases, positive expression for at least one of these antibodies was detected. Because TRKA and TRKB staining was positive in most of our cases including those with negative staining for the pan-TRK antibody, it is worthy to note that using antibodies for TRKA or TRKB could overestimate cases potentially harboring *NTRK* fusions, which is consistent with a previous report [17]. In conclusion, ten ESCC cases showedpositive TRK expression, but none of the GA cases were selected for confirmation analysis to detect *NTRK* fusions.

### 2.2. Confirmation of NTRK Fusion in TRK^+^ ESCC Cases

**Figure 2:**
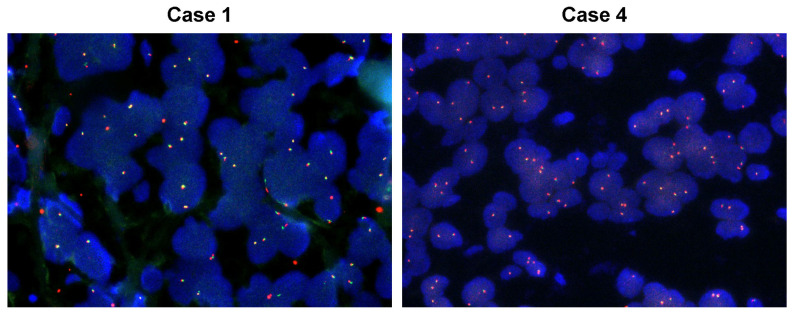
*NTRK3* FISH in esophageal squamous cell carcinoma (ESCC). Representative images from two cases (case 1 and case 4) showing FISH for *NTRK3* fusion.

Ten TRK^+^ ESCC cases were deemed to potentially harbor *NTRK* fusions by IHC screening, and they were subjected to DNA-based NGS analysis known as the NCC Oncopanel. Due to the poor quality of the DNA extracted from FFPE samples, sequencing data were obtained from eight out of ten ESCC cases. As a result, none of *NTRK1* or *NTRK2* fusions were detected in the sequenced ESCC cases. Because *NTRK3* is not included in the NCC Oncopanel, we performed FISH to detect *NTRK3* fusions (Figure 2). After confirming the validity of the experimental methodology using a *NTRK1* fusion positive case, we confirmed that none of *NTRK3* fusions were detected in the ten TRK^+^ ESCC cases (Table 3 and Supplemental Figure S2). In addition, no *NTRK* aberrations, including amplification, were identified in the sequenced ESCC cases. All gene alterations detected in ESCC cases were listed, and the lack of *NTRK* mutation or copy number alterations was confirmed (Table 4 and Supplementary Table S1).

### 2.3. Database NTRK Fusion Detection

Next, we summarized the prevalence of patients with esophagogastric cancer harboring *NTRK* fusions using the TCGA database (n = 3960), and we included esophageal cancer (n = 1476, 968 adenocarcinoma and 508 squamous cell carcinoma), esophagogastric cancer (n = 528 with 140 adenocarcinoma and 388 unknown histology), gastroesophageal junction cancer (n = 268, all adenocarcinoma), and gastric cancer (n = 1672, all adenocarcinoma) (Table 5). Three cases (0.08%, n = 3/3960) expressing *NTRK1* fusions (*RRP15-NTRK1*, *NAV1-NTRK1*, and one case with both *PEAR1-NTRK1* and *NTRK1-STK11* fusions) were detected in esophagogastric adenocarcinoma (esophageal, esophagogastric, or gastroesophageal junction). However, *NTRK* fusions were not detected in patients with ESCC or GA from Western populations. Contrastingly, the China Pan-cancer study (n = 10194) revealed that two of 582 (0.3%) ESCC cases and seven of 850 (0.8%) GA cases from Asian populations expressed *NTRK* fusions (Table 5). The fusion partner of *NTRK3* detected in the China Pan-cancer study is not a common partner detected in other adult *NTRK3* fusion-positive tumors [1,17]. Consistently, a recent real-world data study using cancer comprehensive genomic profiling testing (FoundationOne CDx) further confirmed that *NTRK* fusions are rarely detected in esophageal (0.24%) and gastric (0.16%) cancers [8].

## 3. Discussion

In the present study, we demonstrated that in our cohort of ESCC and GA cases, only ten ESCC cases, and no GA cases, were TRK^+^ on initial IHC screening, and that none of the TRK^+^ ESCC cases subjected to NGS analysis or FISH expressed *NTRK* fusions. The TCGA study revealed that other than the *PEAR1-NTRK1* fusion, which has been detected in patients with breast cancer and sarcoma [17,18], *NTRK* fusions identified in esophageal and gastric cancers are not common mutations. In the TCGA study of Western populations, *NTRK* fusions were detected in patients with esophageal adenocarcinoma and gastroesophageal junction adenocarcinoma but were not detected in ESCC or GA cases. On the other hand, in the TCGA study of Asian populations, *NTRK* fusions were detected in patients with ESCC and GA but were not detected in esophageal adenocarcinoma patients. A case report from Japan described a patient with GA harboring the *ATP1B-NTRK1* fusion, which is also a less common fusion. The *ATP1B-NTRK1* fusion is not listed in other cancers of the TCGA database [19]. Because adenocarcinoma is the most common type of esophageal cancer in Western populations, while squamous cell carcinoma is more common in Asian populations, *NTRK* fusions might not be detected in the small number of Asian patients with esophageal adenocarcinoma [20]. Furthermore, since the overall prevalence of *NTRK* fusion-positive tumors was relatively high in patients with Asian ancestry (0.40%) compared to patients with European ancestry (0.28%), this could have influenced the finding that *NTRK* fusions were more likely to be detected in Asian populations [8]. However, since *NTRK* fusions are extremely rare in ESCC and GA even among Western or Asian populations from the database analyses, it is challenging to study *NTRK* fusions, and this has become a limitation in the research.

The European Society for Medical Oncology (ESMO) established a practical guideline to detect *NTRK* fusions using the molecular diagnosis methods available in daily practice [10]. Although an NGS assay for *NTRK1*, *NTRK2*, and *NTRK3* is the confirmatory method, the performance of NGS analysis for unselected patients is uncommon due to limited NGS access in clinical settings. For tumor types with a high incidence of *NTRK* fusions, FISH, RT-PCR, or RNA-based NGS are recommended to determine if *NTRK* fusions are present. On the other hand, in tumors with low incidence of *NTRK* fusions, TRK detection with IHC screening followed by NGS analysis for detecting *NRTK* fusions is recommended. In the present study, a pan-TRK antibody (EPR17341), which has already been confirmed to be highly sensitive and specific to *NTRK* fusions, was used for IHC screening [11]. Prior studies reported that while TRK expression was observed in gastric gland and tumor-adjacent nerve tissue, TRK was not expressed in the tumor component among 372 Caucasian GA cases [13]. Another study also reported that 7 out of 477 GA tumors were identified as TRK^+^ by IHC, but no *NTRK* fusions were detected by NGS [14]. Furthermore, even though 1 of 66 GA tumors were TRK^+^ by IHC, and the break-apart of *NTRK1* was confirmed by FISH, *NTRK1* fusions were not confirmed by NGS [21]. Importantly, these reports demonstrated that even when positive IHC staining for TRK was detected in ESCC and GA tumors, none of *NTRK* fusions was confirmed by NGS [13,14,21]. A parallel analysis using anti-TRKA, anti-TRKB and anti-TRKC antibodies revealed that positive TRKA and TRKB staining was more frequently detected than anti-pan-TRK staining in ESCC. Based on the NGS finding that none of the TRK^+^ cases harbored *NTRK* fusions, IHC screening with antibodies against TRKA or TRKB could produce relatively high false-positive rates for *NTRK* fusions. Most importantly, since IHC staining for TRK was also negative in our positive control of *NTRK1* fusion-positive esophageal cancer, IHC screening may require further consideration. In conclusion, consistent with previous reports indicating that IHC staining has limited utility as a biomarker for detecting tumors harboring *NTRK* fusions, awaiting the development of more reliable antibodies may help reduce the time and financial burden associated with identifying false-negative *NTRK* fusion cases through NGS [22].

It should be noted that *NTRK* amplifications also induce increased TRK expression, and the prevalence of *NTRK* amplifications has been reported to be 0.48% of AACR GENIE cases, including various types of cancers [23]. While we did not identify *NTRK* amplifications in our ESCC cases, analysis of the TCGA database demonstrated that only one GA case from Asian populations harbored *NTRK1* amplifications. *NTRK1* is located at 1q23.1, and an increased copy number involving that site is considered a potential oncogenic alteration in patients with malignant melanoma and hepatocellular carcinoma, but gene amplification including 1q23.1 is not a common structural variant in esophageal or gastric cancers [24,25].

In this study, *NTRK* fusions are rarely detected in patients with ESCC or GA from Asian populations, and the analyses were reconfirmed. Because ESCC and GA are verified to be tumors with low incidence of *NTRK* fusions, IHC screening followed by NGS confirmation is recommended for detection of *NTRK* fusions. However, IHC staining for TRK could include many false-positive cases, confirmation by NGS would be required prior to considering treatment with TRK inhibitors. The implementation of IHC testing for tumor with low *NTRK* fusion positivity rates may be justified when using TRK antibodies that demonstrate high positive predictive values for TRK. So far, direct NGS testing would likely be indicated only for tumors with high NTRK fusion positivity rates.

## 4. Materials and Methods

### 4.1. Patients

The study included 254 ESCC and 401 GA specimens from patients who underwent surgical resection at Fukushima Medical University Hospital between 2002 and 2021. All cases have been used in our previous studies, and basic characteristics were previously documented [26,27,28]. GA samples were previously subjected to Epstein-Barr virus-encoded small RNA (EBER) ISH to determine EBV infection status and IHC staining for the mismatch repair (MMR) proteins MLH1, MSH2, MSH6, and PMS2 [29]. Data on age, sex, TNM stage (an updated standard for staging cancer, focusing on Tumor (T), Node (N), and Metastasis (M), 8th classification), and pathological diagnosis were retrospectively collected. The carcinomas at the time of primary tumor resection were staged according to the guidelines set forth by the Union for International Cancer Control classification. The study was approved by the ethics committee of Fukushima Medical University (Protocol No. 2367 in 2016 initial approval and REC2024-041 in 2024 renewal, 24 January 2025). For the use of previously collected specimens, the approved research protocol was disclosed in accordance with relevant guidelines, and patients were given the opportunity to opt out. For the prospective collection of specimens, written informed consent was obtained from all participants. All experiments were conducted in accordance with the approved study plan and relevant guidelines.

### 4.2. Immunohistochemical Staining and Evaluation

IHC staining was performed using the polymer peroxidase method with formalin-fixed, paraffin-embedded (FFPE) histology sections (4 μm thick), as described previously [29]. Briefly, the sections were treated for deparaffinization and rehydration, then endogenous peroxidase activity was blocked using 0.3% hydrogen peroxide. After rinsing in PBS, the sections were incubated with ananti-pan-TRK antibody (#EPR17341; 1:50 dilution; Abcam, Cambridge, UK), anti-TRKA antibody (#2510; 12G8; 1:400 dilution; Cell Signaling Technology, Danvers, MA, USA), anti-TRKB antibody (#4607; 80G2; 1:2000 dilution; Cell signaling Technology), or anti-TRKC antibody (#3376; C44H5; 1:1000 dilution; Cell Signaling Technology) at 4 °C overnight. Incubation was performed by a peroxidase-labeled polymer conjugated to goat antirabbit immunoglobulins (ENvision + kit; Dako, Agilent, Santa Clara, CA, USA) as the secondary antibody for 30 min at room temperature, after the PBS wash. For visualization, diaminobenzidine staining was performed, followed by counterstaining with hematoxylin and eosin (H&E). TRK positivity was calculated as the percentage of positive staining cancer cell and defined as negative, 0%; weak positive, <1%; moderate positive, 1–10%; and strong positive, 11–100%, as previously described [29].

### 4.3. Next Generation Sequencing

NGS analysis was performed using the NCC Oncopanel, as described previously [30]. Briefly, extracted genomic DNA from theFFPE section was prepared for sequencing libraries using SureSelect XT reagent (Agilent Technologies, Santa Clara, CA, USA) and a KAPA Hyper Prep kit (KAPA Biosystems, Wilmington, MA, USA). Subsequently, samples were analyzed on the Illumina NextSeq platform (Illumina, San Diego, CA, USA) with 150 bp paired-end reads. The NCC Oncopanel test is a hybridization capture-based NGS assay designed to examine mutations, amplifications, and homozygous deletions of the entire coding region of 124 genes of clinical or preclinical relevance, together with rearrangements of 13 oncogenes, *NTRK1*,and *NTRK2*, included in the panel.

After the adapter sequences using the Cutadapt program was removed, the obtained result of the was mapped to the human reference genome Burrows–Wheeler Aligner and the Smith–Waterman algorithm The cisCall program (version 7.1.5) was used for detected somatic mutations (single nucleotide variants (SNV) and short insertions and deletions (Indels)), copy number amplifications (CNV), and rearrangement (homozygous deletions and fusions). Somatic mutations were selected using the previously described criteria using the NHLBI GO Exome Sequencing Project (ESP6500) (http://evs.gs.washington.edu/EVS/) (accessed on 29 June 2019) or the Integrative Japanese Genome Variation Database (iJGVD, 20181105) (https://ijgvd.megabank.tohoku.ac.jp/) (accessed on 29 June 2019) to remove single nucleotide polymorphisms (SNPs) [30]. Mutations were classified as “pathogenic/likely pathogenic variants” in ClinVar or “oncogenic/likely oncogenic variants” in the OncoKB (http://oncokb.org) databases using oncokb-annotatorand commit 8910b65 (accessed on 29 June 2019). Amplifications with >4-fold copy number increases were defined as positive, and genes with <0.5-fold copy number decreases were defined as homozygous deletion candidates. Somatic mutation and amplification were determined using the Integrative Genomics Viewer (IGV; http://www.broadinstitute.org/igv/ accessed on 29 June 2019).

### 4.4. FISH

FISH analysis of formalin-fixed, paraffin-embedded (FFPE) histology sections (10 μm thick) was performed using the ZytoLight SPEC NTRK3 Dual Color Break Apart Probe (PL164) (ZytoVision GmbH, Bremerhaven, Germany), according to the manufacture’s information. Slides were evaluated for break-apart signals of cancer cells under the fluorescent microscope (Nikon Eclipse Ci with fluorescence LED illumination system D-LEDI) (Nikon, Tokyo, Japan) independently by two pathologists (K. Miyabe and A. Goto) who were blinded to the patient’s history and histological findings.

### 4.5. Database Analysis

Gene change data of patients with esophageal and gastric cancer including the expression of *NTRK* fusions were obtained from the TCGA cBioPortal database (http://www.cbioportal.org/). *NTRK1*, *NTRK2*, and *NTRK3* data were used for the analysis.

## Figures and Tables

**Table 1:** Clinicopathological characteristics of esophageal squamous cell cancer patients.

		Total
Characteristics		(n = 254)
Age-year		
	Mean	66
	Range	37–83
Gender-no. (%)		
	Male	219 (86)
	Female	35 (14)
Race. (%)		
	Asian (Japanese)	254 (100)
Tumor location. (%)		
	Upper	51 (20)
	Middle	130 (51)
	Lower	73 (29)
Histological type-no. (%)		
	well	64 (25)
	moderate	122 (48)
	poor	39 (15)
	Unknown	29 (11)
TNM Stage-no. (%)		
	0	32 (13)
	I	43 (17)
	II	85 (33)
	III	82 (32)
	IV	12 (5)
LN metastasis-no. (%)		
	Positive	140 (55)
	Negative	114 (45)
Lymphatic invasion-no. (%)		
	Present	130 (51)
	Absent	124 (49)
Venous invasion-no. (%)		
	Present	144 (57)
	Absent	110 (43)

**Table 2:** Clinicopathological characteristics of gastric adenocarcinoma patients.

		Total
Characteristics		(n = 401)
Age-year		
	Mean	67.7
	Range	30–92
Gender-no. (%)		
	Male	283 (71)
	Female	118 (29)
Race. (%)		
	Asian (Japanese)	401 (100)
Tumor location. (%)		
	Upper	128 (32)
	Middle	136 (34)
	Lower	124 (31)
	Whole	6 (1)
	N/A	7 (2)
Histological type-no. (%)		
	Differentiated	208 (52)
	Undifferentiated	193 (48)
TNM Stage-no. (%)		
	I	219 (55)
	II	79 (20)
	III	70 (17)
	IV	33 (8)
LN metastasis-no. (%)		
	Positive	247 (62)
	Negative	154 (38)
Lymphatic invasion-no. (%)		
	Present	218 (54)
	Absent	182 (45)
	N/A	1 (0.2)
Venous invasion-no. (%)		
	Present	228 (57)
	Absent	172 (42)
	N/A	1 (0.2)
Mismatch repair (MMR)-no. (%)		
	deficient MMR	33 (8.2)
	proficient MMR	368 (92)
Epstein-Barr virus (EBV)-no. (%)		
	Positive	27 (6.7)
	Negative	374 (93.3)

**Table 3:** Immunohistochemical (IHC) staining confirming by next generation sequencing (NGS) analysis or fluorescence in situ hybridization (FISH) in esophageal squamous cell carcinoma.

	IHC				NGS		FISH
Case number	TRK (pan-TRK)	TRKA	TRKB	TRKC	*NTRK1* fusion	*NTRK2* fusion	*NTRK3* fusion
1	**++**	**+**	**+**	**-**	N.D.	N.D.	N.D.
2	**++**	**-**	**-**	**+**	N.D.	N.D.	N.D.
3	**++**	**-**	**+**	**-**	N.D.	N.D.	N.D.
4	**+**	**+**	**++**	**-**	N.D.	N.D.	N.D.
5	**+**	**+**	**-**	**-**	N.D.	N.D.	N.D.
6	**+**	**-**	**++**	**-**	N.D.	N.D.	N.D.
7	**+**	**-**	**+**	**-**	N.D.	N.D.	N.D.
8	**+**	**-**	**+**	**-**	N.D.	N.D.	N.D.
9	**+**	**+**	**+**	**-**	Failed	Failed	N.D.
10	**+**	**+**	**-**	**-**	Failed	Failed	N.D.
							
Control	**-**	**-**	**-**	**-**	Detected	N.D.	N.D. (*NTRK1* fusion detected)

Staining intensity; (-), negative; (+), weak positive; (++), moderate positive; not detected, N.D.

**Table 4:** Next generation sequencing result in esophageal squamous cell carcinoma.

		Copy Number Alterations	
Case number	Mutations	Amplifications	Homozygous deletions
1	*CDKN2A*, *NFE2L2*, *PALB2*, *TP53*		
2	*TP53*	*FGFR1*	*CDKN2A*, *RB1*
3	*NFE2L2*, *RB1*, *TP53*		
4	*NOTCH1*, *NOTCH3*, *TP53*		*VHL*
5	*KDM6A*, *PIK3CA*, *TP53*	*MYC*	*VHL*, *RHOA*, *RAD51C*
6	*PTCH1*, *TP53*		
7	*EP300*, *PIK3CA*, *TP53*		*RHOA*, *FLT3*
8	*NFE2L2*, *TP53*		

**Table 5:** The prevalence of *NTRK* fusions detected in esophagogastric cancer.

			No. of Patients (%)				
Tumor Sample			Any *NTRK* fusions	*NTRK1* fusion	*NTRK2* fusion	*NTRK3* fusion	Detected *NTRK* fusions
TCGA study							
	Esophagogastric cancer (Exclude China Pan-cancer study)		3/3960 (0.08%)	3	0	0	
		Esophagogastric adenocarcinoma	1/528 (0.19%)	1	0	0	*PEAR1-NTRK1, NTRK1-STK11*
		Adenocarcinoma of the gastroesophageal junction	1/268 (0.37%)	1	0	0	*RRP15-NTRK1*
		Esophageal adenocarcinoma	1/968 (0.10%)	1	0	0	*NAV1-NTRK1*
		Esophageal squamous cell carcinoma	0/508 (0%)	0	0	0	**-**
		Gastric adenocarcinoma	0/1672 (0%)	0	0	0	**-**
	China Pan-cancer study						
		Esophageal adenocarcinoma	0/12 (0%)	0	0	0	**-**
		Esophageal squamous cell carcinoma	2/582 (0.3%)	0	0	2	*NTRK3-NFATC1*, *NTRK3-SEC11A*
		Gastric adenocarcinoma	7/850 (0.8%)	1	1	5	*INSRR1-NTRK1*, *SLC24A2-NTRK2*, *LRRC28-NTRK3*, *LOC100507065-NTRK3*, *Intergenic-NTRK3*, *NTRK3-Intergenic*
FoundationCore database							
	Esophageal cancer		18/7469 (0.24%)	n/a	n/a	n/a	n/a
	Gastric cancer		8/5045 (0.16%)	n/a	n/a	n/a	n/a

Abbreviations: n/a, Not available.

## Data Availability

The datasets generated and/or analyzed during the current study are available from the corresponding author uponreasonable. The original data presented in the study are openly available in the TCGA cBioPortal database (http://www.cbioportal.org/).

## Supplementary Materials

The following supporting information can be downloaded at: https://www.mdpi.com/article/10.3390/ijms27010336/s1.

## Abbreviations

EBER	Epstein-Barr virus-encoded small RNA
EBV	Epstein-Barr virus
ESCC	esophageal squamous cell carcinoma
FISH	fluorescence in situ hybridization
GA	gastric adenocarcinoma
IHC	immunohistochemistry
NGS	next generation sequencing
MMR	mismatch repair
NTRK	Neurotrophic tyrosine receptor kinase
TCGA	The Cancer Genome Atlas
TKI	tyrosine kinase inhibitors
