# Supplementary material for: Evaluation of NTRK Fusions Detection Method in Esophageal Squamous Cell Carcinoma and Gastric Adenocarcinoma
Source: Int J Mol Sci. 2025 Dec 28;27(1):336. doi: 10.3390/ijms27010336 (PMC12786219; doi:10.3390/ijms27010336)
Supplement: Supplementary file 1 [file ijms-27-00336-s001.zip › 251209_NTRK Supple file.pptx]

## Slide 1
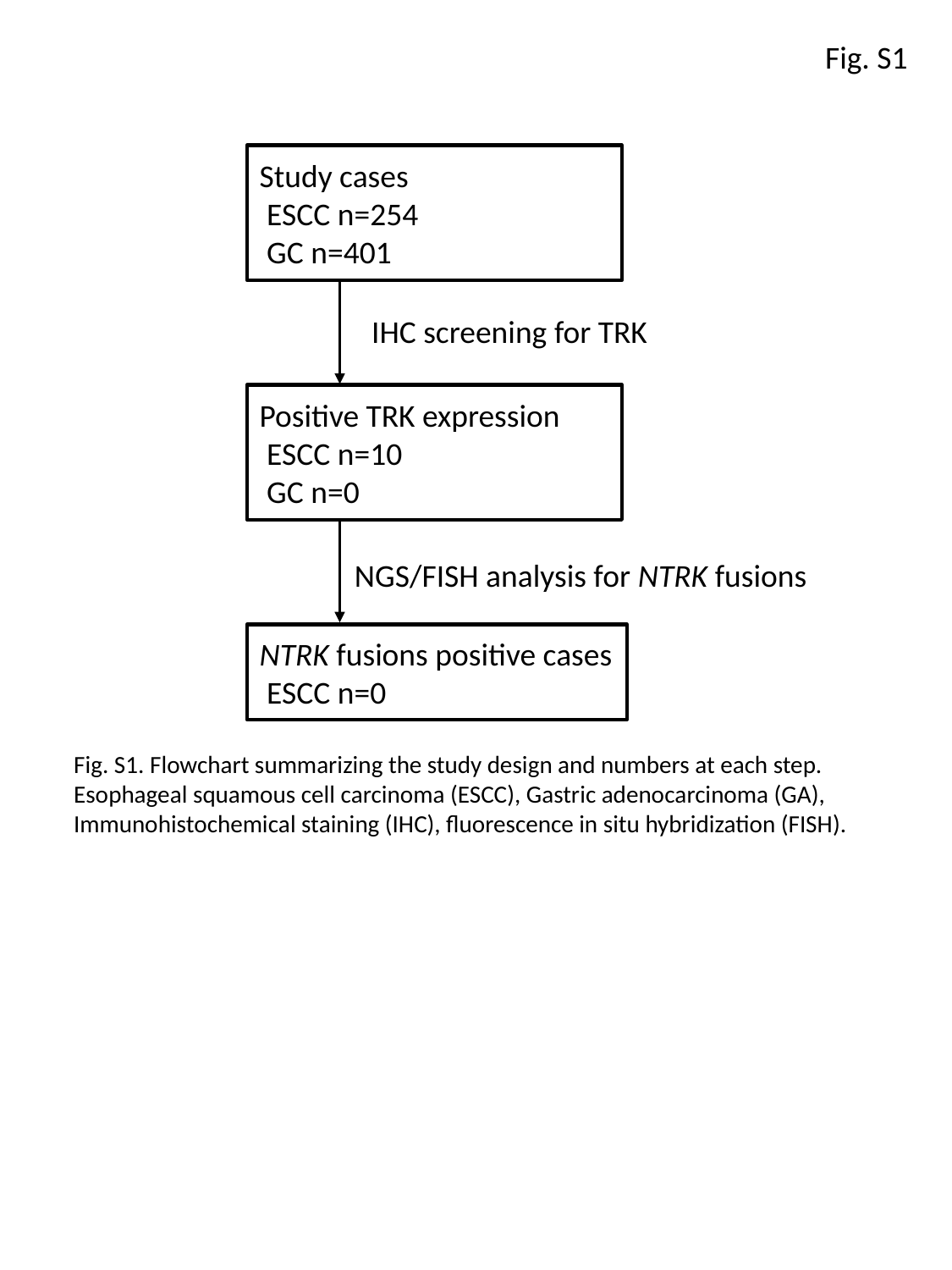

Fig. S1
Study cases
 ESCC n=254
 GC n=401
IHC screening for TRK
Positive TRK expression
 ESCC n=10
 GC n=0
NGS/FISH analysis for NTRK fusions
NTRK fusions positive cases
 ESCC n=0
Fig. S1. Flowchart summarizing the study design and numbers at each step. Esophageal squamous cell carcinoma (ESCC), Gastric adenocarcinoma (GA), Immunohistochemical staining (IHC), fluorescence in situ hybridization (FISH).

## Slide 2
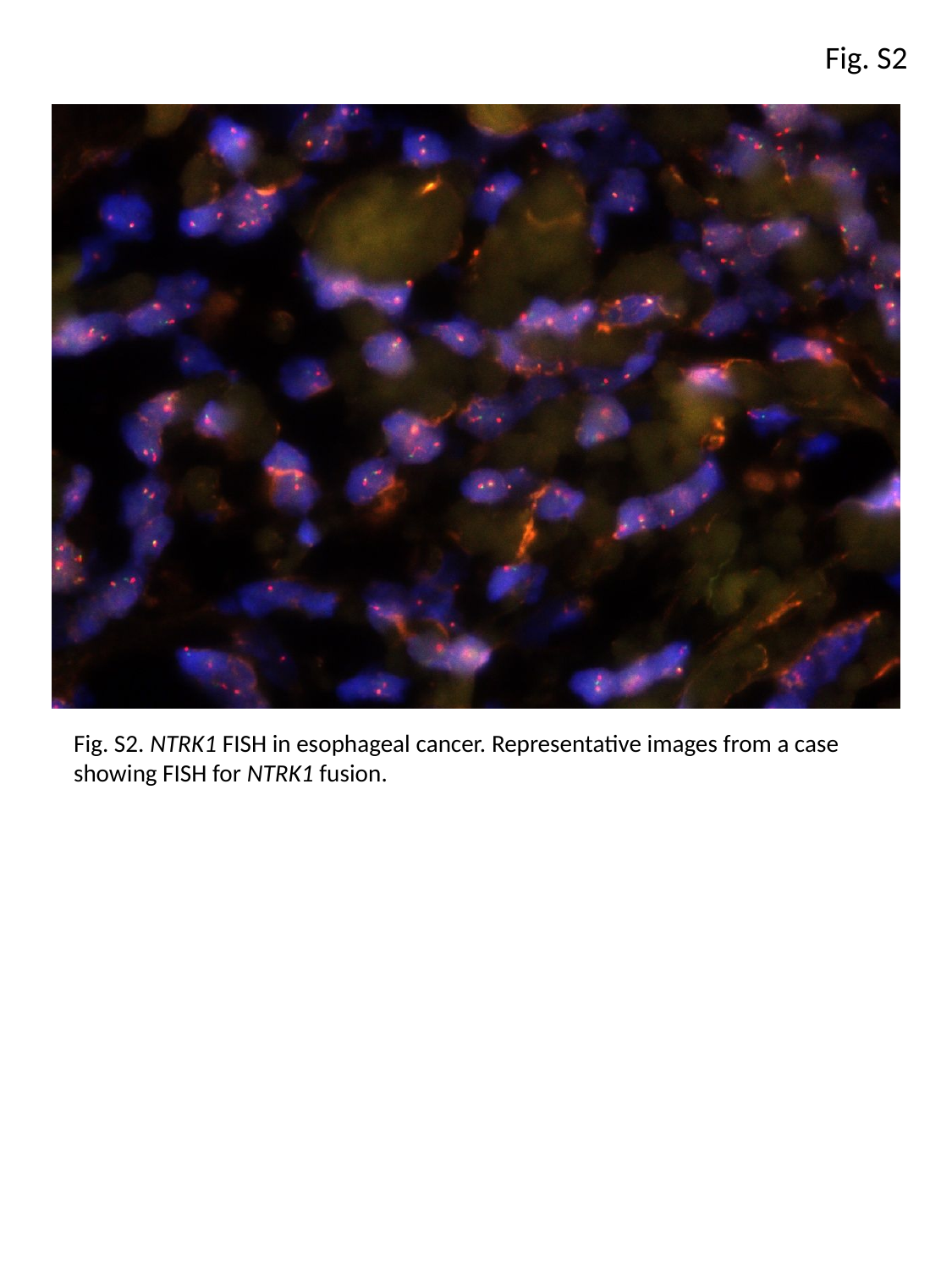

Fig. S2
Fig. S2. NTRK1 FISH in esophageal cancer. Representative images from a case showing FISH for NTRK1 fusion.

## Slide 3
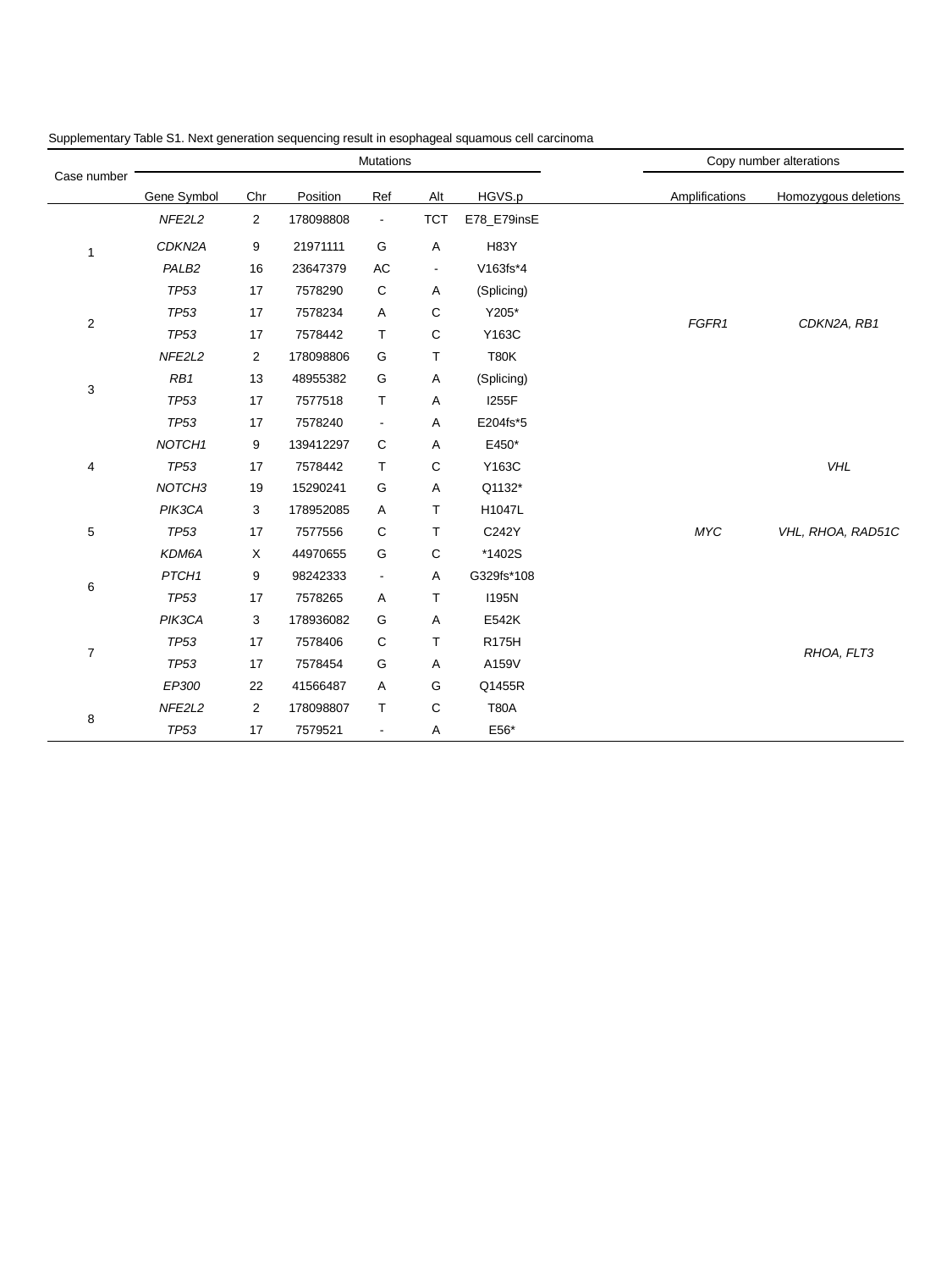

| Supplementary Table S1. Next generation sequencing result in esophageal squamous cell carcinoma | | | | | | | | | |
| --- | --- | --- | --- | --- | --- | --- | --- | --- | --- |
| Case number | | Mutations | | | | | | Copy number alterations | |
| | Gene Symbol | Chr | Position | Ref | Alt | HGVS.p | | Amplifications | Homozygous deletions |
| 1 | NFE2L2 | 2 | 178098808 | - | TCT | E78\_E79insE | | | |
| | CDKN2A | 9 | 21971111 | G | A | H83Y | | | |
| | PALB2 | 16 | 23647379 | AC | - | V163fs\*4 | | | |
| | TP53 | 17 | 7578290 | C | A | (Splicing) | | | |
| 2 | TP53 | 17 | 7578234 | A | C | Y205\* | | FGFR1 | CDKN2A, RB1 |
| | TP53 | 17 | 7578442 | T | C | Y163C | | | |
| 3 | NFE2L2 | 2 | 178098806 | G | T | T80K | | | |
| | RB1 | 13 | 48955382 | G | A | (Splicing) | | | |
| | TP53 | 17 | 7577518 | T | A | I255F | | | |
| | TP53 | 17 | 7578240 | - | A | E204fs\*5 | | | |
| 4 | NOTCH1 | 9 | 139412297 | C | A | E450\* | | | VHL |
| | TP53 | 17 | 7578442 | T | C | Y163C | | | |
| | NOTCH3 | 19 | 15290241 | G | A | Q1132\* | | | |
| 5 | PIK3CA | 3 | 178952085 | A | T | H1047L | | MYC | VHL, RHOA, RAD51C |
| | TP53 | 17 | 7577556 | C | T | C242Y | | | |
| | KDM6A | X | 44970655 | G | C | \*1402S | | | |
| 6 | PTCH1 | 9 | 98242333 | - | A | G329fs\*108 | | | |
| | TP53 | 17 | 7578265 | A | T | I195N | | | |
| 7 | PIK3CA | 3 | 178936082 | G | A | E542K | | | RHOA, FLT3 |
| | TP53 | 17 | 7578406 | C | T | R175H | | | |
| | TP53 | 17 | 7578454 | G | A | A159V | | | |
| | EP300 | 22 | 41566487 | A | G | Q1455R | | | |
| 8 | NFE2L2 | 2 | 178098807 | T | C | T80A | | | |
| | TP53 | 17 | 7579521 | - | A | E56\* | | | |
